# A multiantigenic antibacterial nanovaccine utilizing hybrid membrane vesicles for combating *Pseudomonas aeruginosa* infections

**DOI:** 10.1002/jev2.12524

**Published:** 2024-10-14

**Authors:** Xinran Peng, Yuanjing Luo, Li Yang, Yi Yan Yang, Peiyan Yuan, Xinhai Chen, Guo‐Bao Tian, Xin Ding

**Affiliations:** ^1^ School of Pharmaceutical Sciences (Shenzhen) Shenzhen Campus of Sun Yat‐sen University Shenzhen PR China; ^2^ Bioprocessing Technology Institute (BTI), Agency for Science, Technology and Research (A*STAR) Singapore Singapore; ^3^ Institute of Infectious Diseases, Shenzhen Bay Laboratory Shenzhen China; ^4^ Department of Immunology and Microbiology, Advanced Medical Technology Center, The First Affiliated Hospital, Zhongshan School of Medicine Sun Yat‐sen University Guangzhou China; ^5^ Key Laboratory of Tropical Diseases Control (Sun Yat‐sen University), Ministry of Education Guangzhou China; ^6^ School of Medicine Sun Yat‐Sen University Shenzhen China; ^7^ State Key Laboratory of Anti‐Infective Drug Discovery and Development; School of Pharmaceutical Sciences Sun Yat‐sen University Guangzhou China

**Keywords:** antibacterial, bacterial toxins, hybrid cell membrane, outer membrane vesicle, vaccine

## Abstract

Bacterial infections, especially those caused by multidrug‐resistant pathogens, pose a significant threat to public health. Vaccines are a crucial tool in fighting these infections; however, no clinically available vaccine exists for the most common bacterial infections, such as those caused by *Pseudomonas aeruginosa*. Herein, a multiantigenic antibacterial nanovaccine (AuNP@HMV@SPs) is reported to combat *P. aeruginosa* infections. This nanovaccine utilizes the hybrid membrane vesicles (HMVs) created by fusing macrophage membrane vesicles (MMVs) with bacterial outer membrane vesicles (OMVs). The HMVs mitigate the toxic effects of both OMVs and bacterial secreted toxins (SP) adsorbed on the surface of MMVs, while preserving their stimulating properties. Gold nanoparticles (AuNPs) are utilized as adjuvant to enhance immune response without comprising safety. The nanovaccine AuNP@HMV@SPs induces robust humoral and cellular immune responses, leading to destruction of bacterial cells and neutralization of their secreted toxins. In murine models of septicemia and pneumonia caused by *P. aeruginosa*, AuNP@HMV@SPs exhibits superior prophylactic efficacy compared to control groups including OMVs, or MMVs@SPs and HMV@SPs, achieving 100% survival in septicemia and > 99.9% reduction in lung bacterial load in pneumonia. This study highlights AuNP@HMV@SPs as a safe and effective antibacterial nanovaccine, targeting both bacteria and their secreted toxins, and offers a promising platform for developing multiantigenic antibacterial vaccines against multidrug‐resistant pathogens.

## 1 INTRODUCTION

Bacterial infections induced by ESKAPE pathogens, including ***E*** *nterococcus faecium*, ***S*** *taphylococcus aureus*, ***K*** *lebsiella pneumoniae*, ***A*** *cinetobacter baumannii*, ***P*** *seudomonas aeruginosa*, and ***E*** *nterobacter* species, pose a great threat to public health, as this group of highly drug‐resistant pathogens are resistant to commonly used antibiotics. (Tacconelli et al., 2018) These pathogens are associated with a wide range of infections such as bloodstream infection, pneumonia, and urinary tract infection, leading to high mortality and morbidity. (De Oliveira et al., 2020) Conventional antibiotic treatments have become increasingly ineffective against these pathogens. Moreover, the pipeline of new antibiotics is alarmingly scarce. (Mancuso et al., 2021) To address this urgent need, one promising approach is to develop antibacterial vaccine that could activate the immune system to recognize and kill bacteria. Several vaccines have been clinically used to prevent the occurrence of bacterial infections including *Haemophilus influenzae type b* and *Pneumococcal*. (Jansen et al., 2018) However, there is no available vaccine to prevent ESKAPE‐related infections. (De Oliveira et al., 2020) Taking Gram‐negative bacteria *P. aeruginosa* as an example, numerous vaccines aiming at activating immunity to recognize *P. aeruginosa*‐related antigens including lipopolysaccharide O antigen, flagella proteins or outer membrane proteins were reported, while only several vaccines are currently in clinical trials and no vaccine has been approved for clinical use. (Baker et al., 2020; Grimwood et al., 2015; Merakou et al., 2018) The limited success of the vaccines in clinical trials is mainly due to their low protection efficacy. Notably, the virulence of *P. aeruginosa* is associated with multiple factors, including its surface components like lipopolysaccharide (LPS, also called endotoxin) and polysaccharides that mediate the bacterial adherence to host cells, and its secreted toxins such as type III secretion proteins, elastase, alkaline proteases and hemolysins (phospholipase and lecithinase) that damage host tissues or help the bacteria evade the immune response. (Gauthier et al., 2003; Moradali et al., 2017; Sadikot et al., 2005) Therefore, a multiantigenic vaccine that is able to elicit immune responses not only to kill the bacterial cells, but also to neutralize secreted toxins is more desirable as compared to anti‐*P. aeruginosa* vaccine that aims at a single type of bacterial antigens.

Outer membrane vesicles (OMVs) derived from Gram‐negative bacteria exhibit a similar structure and composition to the bacterial outer membrane, mainly containing outer membrane proteins, lipoproteins, and lipopolysaccharide (LPS). (Gan et al., 2021) Compared to their parent bacteria, OMVs’ nonreplicative nature makes them safer biologicals. (Chheda et al., 2024) Due to their abundant membrane antigens and pathogen‐associated molecular patterns (PAMPs) on the surface, OMVs have gained attention as promising multiantigenic vaccines against bacterial infections. (Rani et al., 2022; Zhang et al., 2018) OMVs‐based vaccines against meningococcal group B disease have been clinically used in Cuba, Norway, and New Zealand. (Micoli & MacLennan, 2020) For OMVs as vaccine against *P. aeruginosa* infection, recent preclinical studies have been focused on the integration of adjuvants with OMVs to enhance specific immune responses or optimizing membrane structure to improve lymph node targeting ability. (Noh et al., 2023; Wu et al., 2022) However, these OMVs‐based vaccines are unable to elicit active anti‐toxin immune responses, thereby lacking the ability to neutralize secreted toxins produced by the bacteria. To broaden the application of OMVs as vaccines, other antigens of interest have been modified on the surface of OMVs through direct chemical conjugation or specific bioconjugation. (Weyant et al., 2023) However, the complex decoration process of the antigens and OMVs is impractical, and the pre‐detoxification of the antigens through heating or chemicalization before linking them to OMVs likely weakens the immune activation function of these antigens. Moreover, a significant limitation for the clinical applications of OMVs‐based vaccines is that they may potentially induce severe systemic inflammatory responses, (Kim et al., 2015; Park et al., 2021) likely attributed to the presence of endotoxins on the surface of OMVs. Therefore, a facile strategy to deliver toxin antigens on the surface of OMVs with attenuated toxicity, while maintaining the potent immunity activation ability of OMVs and toxins, is needed for development of antibacterial vaccine.

**SCHEME 1:**
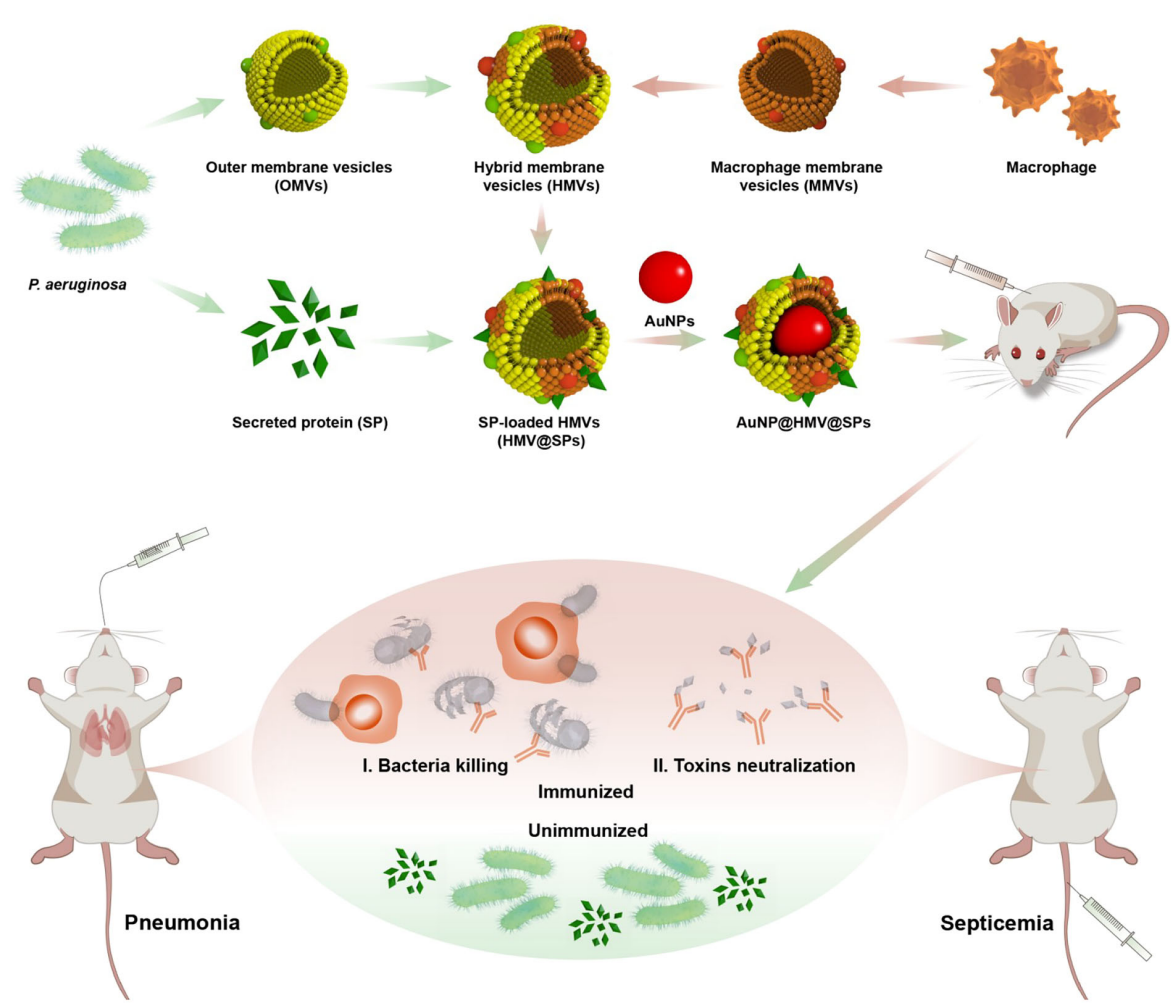
Schematic illustration of the construction and application of multiantigenic hybrid cell membrane‐based vaccine (AuNP@HMV@SPs) against *P. aeruginosa* infection.

In this study, a multiantigenic vaccine (AuNP@HMV@SPs) was developed for the prevention of *P. aeruginosa* infections. The vaccine consists of a hybrid membrane vesicle (HMV@SPs), which is composed of bacterial OMVs and macrophage membrane adsorbed with secreted toxins (SP) of *P. aeruginosa*, and encapsulated with gold nanoparticles (AuNPs) as adjuvant (Scheme 1). The introduction of macrophage membrane vesicles (MMVs) in HMV@SPs serves two purposes. Firstly, they adsorb secreted toxins effectively onto their surface and attenuate the toxicity of the secreted toxins without destroying their structures (Wei et al., 2019), allowing for stimulation of the anti‐toxin immune responses. Secondly, MMVs have been reported to neutralize endotoxins (LPS) through various interactions (Shen et al., 2019), and thus hybridizing MMVs with OMVs also reduces the toxicity of OMVs induced by LPS. Consequently, HMV@SPs could act as a general functional platform that deliver bacterial membrane antigens and secreted antigens simultaneously on its surface with attenuated toxicity, and facilitate immune activation for the clearance of bacterial cells and secreted toxins. To minimize and stabilize the structure of HMV@SPs, small‐sized AuNPs of approximately 30 nm were chosen as an adjuvant (Gao et al., 2015). This further enhances the immune responses against *P. aeruginosa* infection. The antibacterial immunity of AuNP@HMV@SPs was validated in a *P. aeruginosa*‐induced septicemia model (systemic infection) and a *P. aeruginosa*‐induced lung infection model (local infection), and the in vivo activation of T cells and B cells immunity by the vaccine was also explored.

## 2 MATERIALS AND METHODS

### 2.1 Materials

Chloroauric acid was obtained from Xiya Reagent, Shandong, China. Sodium citrate dihydrate was obtained from Macklin, Shanghai, China. Ammonium sulfate was obtained from Tianjin Zhiyuan Chemical Reagent Co., Ltd., Tianjin, China. The bicinchoninic acid (BCA) assay kit, SDS‐PAGE kit, 3‐(4,5‐dimethylthiazol‐2‐yl)‐2,5‐diphenyltetrazolium bromide (MTT) kit, 1,1′‐dioctadecyl‐3,3,3′,3′‐tetramethylindocarbocyanine perchlorate (DiI) and 3,3′‐dioctadecyloxacarbocyanine perchlorate (DiO) were obtained from Beyotime Biotechnology, Shanghai, China. Fluorescein isothiocyanate (FITC) was purchased from Aladdin, Shanghai, China. Mueller–Hinton broth (MHB) was obtained from BD Difco, U.S.A.. CN agar was obtained from Hopebio, Qingdao, China. RPMI 1640 and DMEM cell culture medium were obtained from Gibco, Termo Fisher Scientifc, U.S.A. IL‐6, TNF‐α and IL‐1β ELISA kits were obtained from NeoBioscience Co., Ltd. Shenzhen, China. FITC‐conjugated anti‐mouse CD11c, PE‐conjugated anti‐mouse CD80, APC‐conjugated anti‐mouse CD86, APC‐conjugated anti‐mouse CD3, PE‐conjugated anti‐mouse CD8a, FITC‐conjugated anti‐mouse CD4, and PE‐conjugated anti‐mouse CD19 were purchased from Tonbo Biosciences, U.S.A.. APC‐conjugated anti‐mouse GL‐7 was obtained from Laizee Biotech, Shanghai, China. FITC‐conjugated anti‐mouse IgD was obtained from eBioscience, U.S.A.. Goat Anti‐Mouse IgG was bought from Huayun biotech Co., LTD., Guangzhou, China. Murine IL‐4, murine GM‐CSF were bought from PeproTech, USA. *P. aeruginosa* (PA17ZC010) was clinically isolated from hematology department, and its antibiogram was attached in Table S1. RAW 264.7 cells (ATCC No. TIB‐71) were obtained from American Type Culture Collection (ATCC). LPS ELISA kit was bought from adsbio, Jiangsu, China.

### 2.2 Isolation of *P. aeruginosa*‐derived OMVs, SP, and macrophage‐derived MMVs

OMVs, SP and MMVs were isolated by following the methods described in previous studies. (Li et al., 2023; Wei et al., 2019; Wu et al., 2021) A single colony of *P. aeruginosa* (PA17ZC010) was cultivated in MHB medium at 37°C under shaking at 220 rpm for 18–24 h. The resulting *P. aeruginosa* suspension was centrifuged at 8000 × *g* for 5 min to remove bacteria. Subsequently, the supernatant was ultracentrifuged at 100,000 × *g* for 1 h at 4°C. The supernatant and precipitate were collected for the isolation of SP and OMVs, respectively. For the isolation of SP, ammonium sulfate was added slowly to the supernatant until saturation and stirred overnight at 4°C. The solution was then centrifuged at 4000 × *g* for 1 h to obtain SP. SP was dissolved in PBS and further purified by dialysis (3 kDa) for 4 h. For the isolation of OMVs, the precipitate obtained from ultracentrifugation was washed three times with PBS and resuspended in PBS and stored at ‐80°C.

RAW 264.7 cells were collected and suspended in a hypotonic lysing buffer containing 30 mM Tris‐HCl (pH 7.5), 225 mM d‐mannitol, 75 mM sucrose, 0.2 mM EDTA, and a protease inhibitor cocktail. Subsequently, RAW 264.7 cells were disrupted using a Dounce homogenizer with a tight‐fitting pestle (30 passes). The homogenized suspension was centrifuged at 3000 × *g* for 10 min to remove cell debris with a large size. Then the supernatant was centrifuged again at 20,000 × *g* for 1 h at 4°C to obtain MMVs pellet. Finally, MMVs were washed twice with PBS, suspended in PBS, and stored at ‐80°C.

### 2.3 Preparation and characterization of hybrid membrane vesicles (HMVs) of OMVs and MMVs

OMVs and MMVs were mixed and sonicated (25 kHz, 720 W) for 10 min to form hybrid membrane vesicles HMVs. DiO (excitation/emission = 484/501 nm) and DiI (excitation/emission = 550/567 nm) were used to conduct the Förster resonance energy transfer (FRET) study. MMVs were added to the DiO/DiI‐stained OMVs at MMVs to OMVs protein weight ratios of 0:1, 2:1, and 4:1, respectively. The mixture was then sonicated for 10 min to facilitate membrane fusion. The fluorescence spectrum of each sample was recorded using a fluorescence spectrophotometer (FL‐970, Techcomp, China) with an excitation wavelength of 490 nm.

To evaluate the in vitro immune toxicity of HMVs, the level of cytokines released from RAW 264.7 cells was tested. RAW 264.7 cells were seeded into 24‐well plates (5×10^4^ cells per well) and treated with HMVs at different ratios of MMVs to OMV (OMVs: 2 µg/mL) for 12 h. The levels of IL‐6 and TNF‐α in the supernatants were measured using ELISA kits.

For transmission electron microscopy observation, a drop of samples was precipitated on a carbon‐coated grid, stained with 1% phosphotungstic acid and observed using a FEI Tecnai G2 Spirit TEM (FEI, U.S.A.). Moreover, the membrane fusion was confirmed through observation under a confocal laser scanning microscope (CLSM) (Zeiss, LSM880, Germany). MMVs and OMVs were stained with DiO (Green) and DiI (Red), respectively. HMVs obtained through sonication of the mixture of DiI‐labeled OMVs and DiO‐labeled MMVs were imaged using the CLSM (Zeiss, LSM880, Germany).

### 2.4 Preparation and characterization of SP‐loaded HMVs (HMV@SPs)

HMV@SPs were formed by incubating HMVs with SP at 37°C for 15 min. The hemolytic activity of HMV@SPs was assessed by diluting the whole blood of ICR mice 25‐fold with PBS and incubating the diluted blood with HMV@SPs at different weight ratios of HMVs to SP (Concentration: 80 µg/mL SP) at 37°C for 0.5 h. PBS‐treated blood and 0.1% triton‐treated blood were used as negative control and positive control, respectively. The samples were then centrifuged at 455 × *g* for 5 min and hemolysis was determined by measuring the absorbance of the supernatant at 570 nm using a microplate reader (Thermo Scientifc, Multiskan FC, U.S.A.).

The cytotoxicity of HMV@SPs was analyzed by incubating RAW 264.7 cells with HMV@SPs at different weight ratios of HMVs to SP (Concentration: 80 µg/mL SP) for 24 h. The untreated cells were used as the 100% viability control. MTT solution (100 µL, 0.5 mg/mL in DMEM) was added to different groups of cells and incubated in dark for 4 h, followed by incubating with formazan solution (100 µL) for 4 h. Finally, the absorbance at 570 nm was obtained using a microplate reader (Thermo Scientifc, Multiskan FC, USA).

The in vitro immune toxicity of HMV@SPs was investigated by incubating RAW 264.7 cells with HMV@SPs (Concentration: 2 µg/mL OMVs) for 12 h. The levels of IL‐6 and TNF‐α in the supernatants were measured by using ELISA kits. To evaluate the in vivo immune toxicity of HMV@SPs, mice were injected intraperitoneally with OMVs, SP, or HMV@SPs (Concentration: 5 µg/mL OMVs) and the corresponding serum was collected at 5 h post injection to measure IL‐6 and TNF‐α levels using ELISA kits.

### 2.5 Preparation and characterization of AuNP@HMV@SPs

AuNPs were prepared using a citrate reduction method, as described in a previous report. (Chen et al., 2021) In brief, HAuCl_4_ (3 mg) was dissolved in double distilled water (ddH_2_O, 30 mL) at 95°C. Then, sodium citrate (30 mg) was dissolved in ddH_2_O (0.3 mL) and added to the HAuCl_4_ solution under stirring at 500 rpm. After heating for 30 min, the solution was stirred at room temperature for 30 min, and the resulting solution containing AuNPs was stored at 4°C. To prepare AuNP@HMV@SPs, AuNPs were washed twice with ddH_2_O, and then 200 µg of HMV@SPs was mixed with 100 µg of AuNPs. The mixture was extruded through a 50 nm polycarbonate membrane using an Avanti mini‐extruder.

AuNP@HMV@SPs were observed under TEM following the same method as described in Section 2.3. Hydrodynamic size and surface zeta potential were measured by dynamic light scattering (DLS) on a Zetasizer Nano ZS (Malvern). The LPS contents of AuNP@HMV@SPs, OMVs, SP, MMVs, and HMV@SPs (OMVs: 2 µg, SP: 1.5 µg and MMVs: 4 µg) were measured using ELISA kit. Hemolysis assay and cytotoxicity assay were performed following the same method as described in Section 2.4.

### 2.6 In vitro BMDCs maturation assays

BMDCs generated from bone marrow cells were obtained from male ICR mice (6–8 weeks) through induced differentiation. Briefly, after euthanizing the mice, the cells in femurs and tibias were collected by gently flushing with PBS and washed twice by centrifugation at 300 × *g* for 5 min. Subsequently, the collected cells were cultured in RPMI 1640 medium supplemented with 10% FBS, 2 mM L‐glutamine, 100 µg/mL penicillin, 100 µg/mL streptomycin, 10 ng/mL murine IL‐4, and 20 ng/mL murine GM‐CSF. The cell culture medium was refreshed on day 3 and 5. On day 6, BMDCs were collected for further study.

The cellular uptake of AuNP@HMV@SPs was assessed by incubating BMDCs in confocal dishes (35 mm) for 24 h. Then, BMDCs were washed with PBS and incubated with dye‐labelled AuNP@HMV@SPs (Concentration: 30 µg/mL OMVs) for 1 h or 3 h. After incubation, the cells were washed three times with ice‐cold PBS followed by staining with Hoechst for 10 min. Finally, BMDCs were washed three times with ice‐cold PBS, and imaged using CLSM.

To investigate the effect of AuNP@HMV@SPs on the maturation of BMDCs, BMDCs were incubated in 12‐well plates with fresh medium. AuNP@HMV@SPs (Concentration: 2 µg/mL OMVs) were added into each well and cultured for 24 h at 37°C. The cells were then collected by centrifugation at 300 × g for 5 min and resuspended in PBS. FITC‐conjugated anti‐mouse CD11c, PE‐conjugated anti‐mouse CD80, and APC‐conjugated anti‐mouse CD86 were used for fluorescence labeling, and the labeled cells were analyzed using a flow cytometer (CytoFLEX, Beckman, U.S.A.).

### 2.7 In vivo biocompatibility of AuNP@HMV@SPs

ICR mice were subcutaneously immunized with AuNP@HMV@SPs (containing 2 µg OMVs) on day 0, followed by booster doses on day 7 and day 14. On day 21, serum was collected for biochemical analysis including alkaline phosphatase (ALT), aspartate aminotransferase (AST), blood urea nitrogen (UREA), creatinine (CREA), to evaluate liver and kidney functions. Heart, liver, lung, spleen, and kidney tissues were collected and fixed with 4% paraformaldehyde. Histological analysis of these heart, liver, lung, spleen, and kidney tissues was performed using H&E staining.

### 2.8 In vivo immunization assays of AuNP@HMV@SPs

ICR mice (6–8 weeks) were immunized via subcutaneous injection of AuNP@HMV@SPs (containing 2 µg OMVs) on days 0, 7, and 14. On day 21, serum, lymph nodes (LNs), and spleen were collected for immunization assays.

To evaluate IgG titers, a 96‐well plate was coated with 100 ng of *P. aeruginosa* lysate, OMVs or SP overnight and washed with PBS for three times. Next, the plates were blocked with 2 wt% BSA for 1 h and washed with PBS for three times. Subsequently, serially diluted serum samples were added to each well and incubated for 1 h. After incubation, the plate was washed with PBS for three times followed by incubating with HRP‐conjugated goat anti‐mouse IgG (1:5000) for 1 h. The TMB‐ELISA substrate was then added and incubated for 10 min. The reaction was terminated by the addition of 1 M HCl. Finally, titers were quantified by measuring absorbance at 450 nm.

To evaluate the maturation of DCs, the collected LNs were ground by a syringe piston in an ice bath. A single cell suspension was obtained via passing through a 70 µm cell strainer and washed once with PBS. The samples were stained with FITC‐conjugated anti‐mouse CD11c, PE‐conjugated anti‐mouse CD80, and APC‐conjugated anti‐mouse CD86 for 30 min at 4°C. Flow cytometry data was acquired on a CytoFLEX flow cytometer (Beckman, USA).

To evaluate the frequency of germinal center B cells and T cells, the spleens from inoculated mice were ground and individual spleen cells were obtained using a similar method. Half of the samples were stained with PE‐conjugated anti‐mouse CD19, FITC‐conjugated anti‐mouse IgD, and APC‐conjugated anti‐mouse GL7 for 30 min at 4°C for the determination of the frequency of germinal center B cells. Another half of the cell samples were stained with APC‐conjugated anti‐mouse CD3, PE‐conjugated anti‐mouse CD8a, and FITC‐conjugated anti‐mouse CD4 for 30 min at 4°C for the determination of the frequency of T cells and their subsets. Flow cytometry data was acquired on a CytoFLEX flow cytometer (Beckman, U.S.A.).

### 2.9 Prophylactic effects of AuNP@HMV@SPs against bacterial infection

ICR mice (6–8 weeks) were immunized via subcutaneous injection of AuNP@HMV@SPs (containing 2 µg OMVs) on days 0, 7, and 14. On day 21, the mice were infected with *P. aeruginosa*. A septicemia model was established via the intravenous injection of *P. aeruginosa* (1 × 10^7^ CFU/20 g). At 24 h post infection, five mice were randomly selected and their blood, spleen, lung, and kidney were collected. The tissues were homogenized and the homogenates were plated for CFU counting. In addition, serum samples were also collected to measure the level of IL‐6, TNF‐α, and IL‐1β. On day 25, the remaining mice were euthanized, and the heart, liver, spleen, lung, and kidney tissue were collected for H&E staining. For the survival assessment, the mice were infected intravenously with *P. aeruginosa* at a dose of 1 × 10^7^ CFU/20 g or 2 × 10^7^ CFU/20 g, and their survival rate was monitored daily.

A pneumonia mouse model was established via intratracheally administrating *P. aeruginosa* (1 × 10^7^ CFU/20 g). At 24 h post infection, five mice were randomly selected and their lungs were collected and homogenized. The homogenized suspensions were diluted by PBS and plated for CFU counting. The homogenized suspensions were also utilized to measure the levels of IL‐6, TNF‐α, and IL‐1β by using ELISA kits. Lung tissues were also collected for Gram staining. On day 25, the remaining mice were euthanized, and their lungs was collected for H&E staining.

### 2.10 Statistical analysis

The significant difference was analyzed by Student's *t*‐test and or One‐way ANOVA with Tukey's post hoc test for comparison of two groups or multiple groups. The experiments in this study were performed at least in triplicates, and the results were presented as mean ± SD. Significance was indicated by ns (non‐significance), *p < 0.05, **p < 0.01, ***p < 0.001. All data were analyzed by SPSS Software.

## 3 RESULTS AND DISCUSSION

### 3.1 Preparation and characterization of HMV@SPs

**FIGURE 1:**
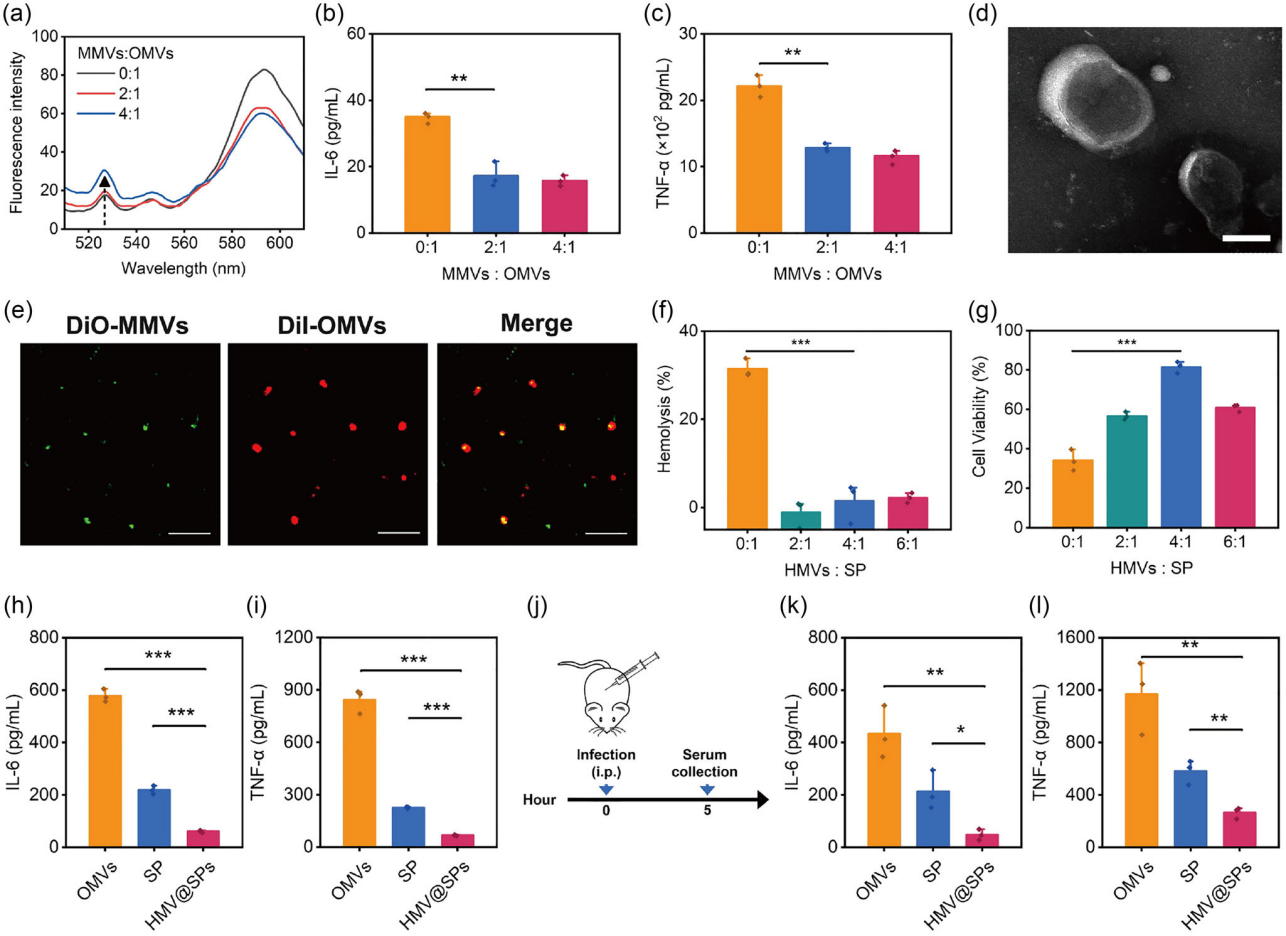
Characterization and toxicity evaluation of HMV@SPs. (a) Fluorescent spectra of OMVs labelled with a FRET dye pair (DiO and DiI) after sonication with unlabeled MMVs at different protein weight ratios. Levels of pro‐inflammatory cytokines (b) IL‐6 and (c) TNF‐α production from RAW 264.7 cells treated with HMVs at different protein weight ratios of MMVs to OMVs for 12 h (2 µg/mL OMVs, *n* = 3). (d) TEM images of HMVs (Scale bar = 100 nm). (e) Fluorescence colocalization images of HMVs fused by DiI‐labelled OMVs and DiO‐labelled MMVs (Scale bars = 3 µm). (f) RBC hemolysis level and (g) cytotoxicity toward RAW 264.7 cells of HMV@SPs at different protein weight ratios of HMVs to SP (80 µg/mL SP, *n* = 3). Levels of pro‐inflammatory cytokine (H) IL‐6 and (I) TNF‐α produced from RAW 264.7 cells treated with OMVs, SP, or HMV@SPs for 12 h (OMVs: 2 µg/mL, SP: 1.5 µg/mL, *n* = 3). (J) Schematic illustration of the experiment to evaluate the in vivo inflammatory toxicity of HMV@SPs. Mice were injected intraperitoneally with OMVs, SP or HMV@SPs, and serum samples were collected at 5 h after injection for measurement of pro‐inflammatory cytokines levels. Serum level of (K) IL‐6 and (L) TNF‐α (OMVs: 5 µg/mL, SP: 3.8 µg/mL, *n* = 3). Statistical analysis was conducted using the student's *t*‐test. *p < 0.05, **p < 0.01, ***p < 0.001.

MMVs and OMVs were isolated from a macrophage cell line RAW 264.7 and a clinically isolated *P. aeruginosa* strain, respectively. To confirm the fusion process of MMVs and OMVs, a Förster resonance energy transfer (FRET) assay was performed. In this assay, a FRET dye pair consisting of 1,1′‐dioctadecyl‐3,3,3′,3′‐tetramethylindocarbocyanine perchlorate (DiI) and 3,3′‐dioctadecyloxacarbocyanine perchlorate (DiO) were utilized to label the OMVs. Subsequently, the labeled OMVs were sonicated with unlabeled MMVs at different protein weight ratios. As the amount of MMVs increased, the fluorescence emission of DiO was increased, while the fluorescence emission of DiI reduced (Figure 1a). The change in the fluorescence indicated the successful hybridization of OMVs and MMVs (HMVs), as it suggested an increase in the distance between the dyes on the OMVs after their interaction with MMVs. Next, we evaluated the in vitro inflammatory toxicity of HMVs at different protein weight ratios of MMVs to OMVs (Figure 1b and 1c). The levels of pro‐inflammatory cytokines IL‐6 and TNF‐α produced by macrophages (RAW 264.7 cells) were significantly reduced when the ratio of MMVs to OMVs was 2:1 and 4:1 as compared to using OMVs alone. This finding indicated that MMVs effectively attenuated the inflammatory toxicity of OMVs. The attenuated toxicity may be attributed to re‐binding of virulence factors such as LPS with receptors on MMVs, which subsequently prevented their stimulation of RAW 264.7 cells. Considering the toxicity and antigen abundance of OMVs, HMVs were formed by sonication of a mixture of MMVs and OMVs at a 2:1 protein weight ratio in the subsequent studies. Under this condition, the obtained HMVs displayed a typical exosome morphology with a diameter of approximately 200 nm (Figure 1d). The fluorescence overlaps of DiI‐labelled OMVs (red) and DiO‐labelled MMVs (green) also conformed the successful construction of HMVs (Figure 1e). Consistent with the inflammatory toxicity assay, HMVs showed lower cytotoxic activity towards RAW 264.7 cells compared to OMVs in vitro (Figure S1). This finding suggests that HMVs have potential in overcoming the issue of toxicity associated with OMVs‐based vaccines.

To enhance the abundance of antigens, secreted proteins from *P. aeruginosa* (SP) were incorporated into HMVs. SP, which contain multiple exotoxins, were isolated using salting out method from the supernatant of *P. aeruginosa* culture broth after removing bacterial cells and OMVs with large particle size. (Wei et al., 2019) SP exhibited significant hemolytic activity (> 20% hemolysis level) and caused severe cytotoxicity (< 30% cell viability) when the concentration of SP reached 80 µg/mL or above (Figure S2 and S3). The toxic nature of SP limited its application as vaccine against infection. However, macrophage membrane can effectively recognize and adsorb secreted toxin derived from bacteria. (Wei et al., 2019) The SP‐neutralization capacity of HMVs was evaluated. After incubating with HMVs, the hemolytic activity of SP was completely neutralized at a low protein ratio (HMVs: SP = 2:1), indicating that SP could be adsorbed onto the surface of HMVs, significantly inhibiting the RBC lysis activity of SP (Figure 1f). Pre‐incubation with HMVs also reduced the cytotoxicity of SP toward RAW 264.7 cells, resulting in over 80% cell viability at a protein ratio of 4:1 (HMVs: SP) (Figure 1g). However, a higher protein ratio (HMVs: SP = 6:1) led to enhanced cytotoxicity, likely due to the toxicity of OMVs at this concentration. Consequently, a protein ratio of 4:1 was chosen for HMVs to SP to form HMV@SPs for subsequent studies.

We further investigated the inflammatory toxicity of HMV@SPs both in vitro and in vivo. After integrating OMVs with SP by the hybrid cell membrane‐based vesicles, HMV@SPs significantly reduced the production of pro‐inflammatory cytokines IL‐6 and TNF‐α from RAW 264.7 cells compared to OMVs and SP (Figure 1h and 1i). More importantly, HMV@SPs induced low expression of IL‐6 and TNF‐α in serum after the intraperitoneal injection in vivo, while the administration of OMVs or SP triggered a potent systemic immune response with substantial release of pro‐inflammatory cytokines (Figure 1j‐1l). HMV@SPs induced significantly lower levels of inflammatory responses compared to OMVs. This is likely because LPS in OMVs binds to the MMVs (Shen et al., 2019), rather than being degraded by components present in MMVs (Figure S4). Collectively, these results suggested that HMV@SPs could serve as a more biocompatible vaccine than OMVs or SP alone.

### 3.2 Preparation and characterization of AuNP@HMV@SPs

**FIGURE 2:**
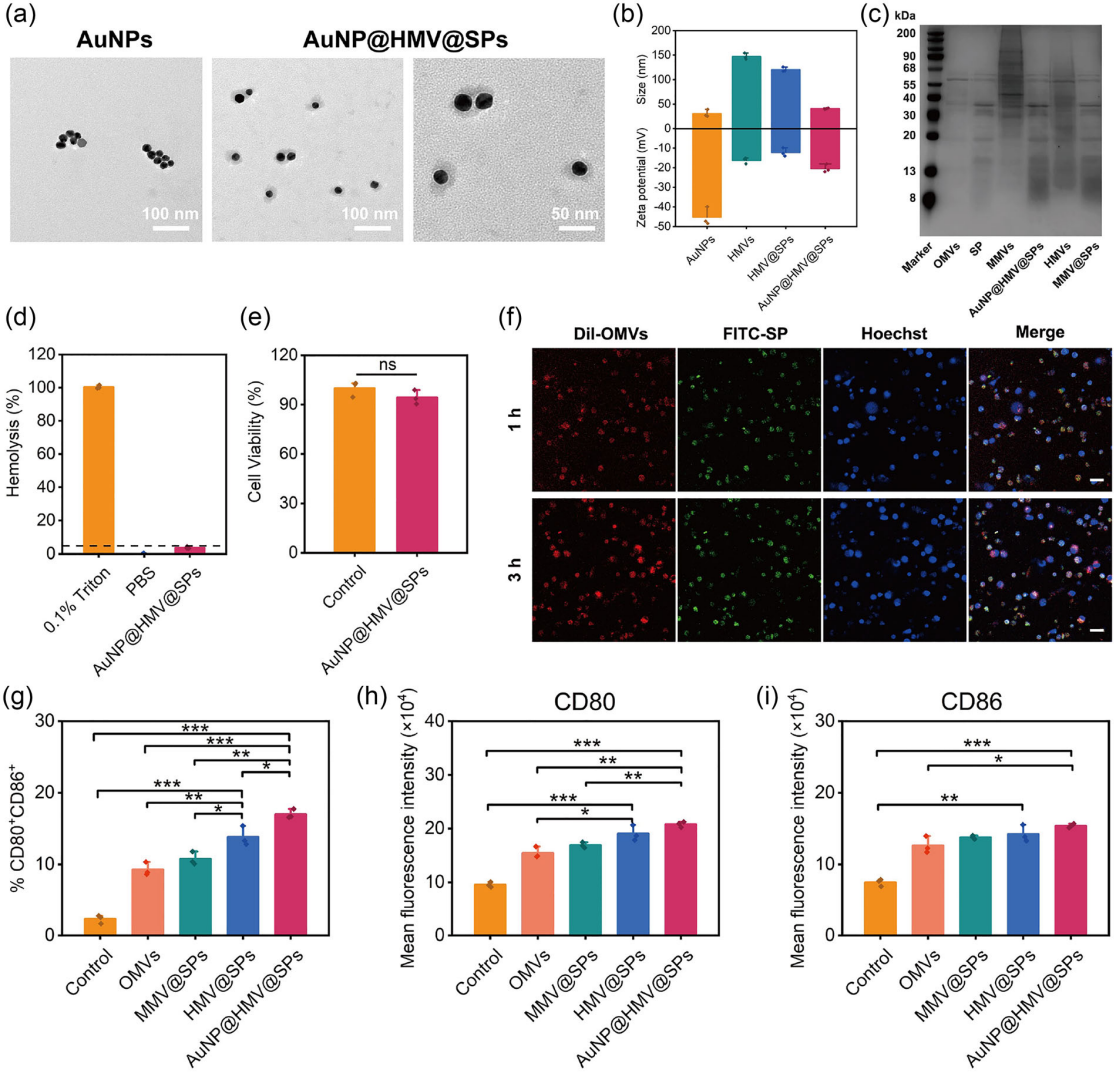
Physiochemical characterization, in vitro toxicity and BMDCs activation of AuNP@HMV@SPs. (a) TEM images of AuNPs and AuNP@HMV@SPs. (b) Hydrodynamic size and zeta potential of AuNPs, HMVs, HMV@SPs, and AuNP@HMV@SPs (*n* = 3). (c) SDS‐PAGE protein analysis of OMVs, SP, MMVs, AuNP@HMV@SPs, HMVs, and MMV@SPs. (d) RBC hemolysis level and (E) Cytotoxicity level towards RAW 264.7 cells of SP, HMV@SPs, and AuNP@HMV@SPs (Concentration: 80 µg/mL SP, *n* = 3). The dashed line represented 5% hemolysis level. (f) CLSM images of BMDCs cultured with AuNP@HMV@SPs for 1 h or 3 h, where DiI (red) and FITC (green) were used to stain OMVs and SP, respectively (30 µg/mL OMVs and 22 µg/mL SP, Scale bars = 20 µm). (g) Percentage of CD80^+^CD86^+^ cells in BMDCs (CD11c^+^ cells) after different treatments for 24 h, and mean florescence intensity of (H) PE‐labeled CD80 and (I) APC‐labeled CD86 in BMDCs (OMVs: 2 µg/mL, SP: 1.5 µg/mL, *n* = 3). Statistical analysis was conducted using the student's *t*‐test. *p < 0.05, **p < 0.01, ***p < 0.001 and ns representing non‐significance.

HMV@SPs were then coated onto AuNPs (AuNP@HMV@SPs) through extrusion, where AuNPs were prepared by citrate reduction (Chen et al., 2021). TEM revealed that AuNP@HMV@SPs possessed a spherical core‐shell structure, indicating the successful camouflage of AuNPs by HMV@SPs (Figure 2a). The increased diameter (41 nm) and zeta potential (‐20 mV) of AuNP@HMV@SPs compared to AuNPs, further confirming the successful coating of HMV@SPs onto the AuNPs (Figure 2b). Importantly, the size of AuNP@HMV@SPs is more uniform than HMV@SPs, as evidenced by the smaller polydispersity index (PDI) of AuNP@HMV@SPs (Figure S5). The AuNPs adjuvant addresses the issue of large size distribution of natural membrane vesicles. Moreover, AuNP@HMV@SPs inherited most proteins of OMVs and SP (Figure 2c), suggesting that multiple antigens from bacterial cell membrane and secretion could be co‐delivered by AuNP@HMV@SPs. Proteomic analysis also confirmed that AuNP@HMV@SPs not only contained proteins from OMVs including flagellin, lipoprotein and outer membrane protein, but also contained proteins from SP including aminopeptidase, elastase and Type III secretion outer membrane protein (Table S2). Despite the protein inheritance, AuNP@HMV@SPs exhibited non‐hemolytic activity and cytocompatibility toward macrophages (Figure 2d and 2e), which is similar to HMV@SPs (Figure 1f and 1g). In addition, both SDS‐PAGE results (Figure 2c) and proteomic analysis (Table S2) showed less MMVs proteins present in AuNP@HMV@SPs, which could be due to the MMVs protein degradation caused by various proteases such as elastase lasA and lasB present in both OMVs and SP. The inclusion of AuNPs adjuvant does not affect the safety of HMV@SPs, and thus AuNP@HMV@SPs could be a safe vaccine to deliver antigens to immune cells.

The activation of bone marrow‐derived dendritic cells (BMDCs) by AuNP@HMV@SPs, which is favorable for immune activation of specific T cells and B cells and prophylactic protection against *P. aeruginosa* infection, was investigated. To evaluate the uptake of antigens on AuNP@HMV@SPs by BMDCs, dyes‐labeled AuNP@HMV@SPs (DiI‐labeled OMVs and FITC‐labeled SP) were incubated with Hoechst‐labelled BMDCs. Confocal laser scanning microscopic (CLSM) images showed colocalized fluorescent signals of DiI and FITC in BMDCs, with the fluorescent intensity increasing as incubation time increased, indicating efficient uptake of AuNP@HMV@SPs by BMDCs (Figure 2f). The uptake of antigens further maturated BMDCs for the activation of specific immune responses. The maturation of BMDCs was characterized by analyzing the expression of co‐stimulatory molecules CD80 and CD86 on the surface of BMDCs (Figure 2g‐2i and S6). Owing to the delivery of multiple antigens and a stable structure with a small size, the treatment with AuNP@HMV@SPs showed the highest percentage of CD80^+^CD86^+^ cells in BMDCs, which increased from 2% to 17% compared to OMVs, MMV@SPs, or HMV@SPs without AuNPs (Figure 2g). The increased CD80^+^CD86^+^ cell population demonstrated that AuNP@HMV@SPs possessed a more potent activation of BMDCs maturation. The highest mean fluorescence intensity of PE‐labeled CD80 and APC‐labeled CD86 in AuNP@HMV@SPs‐treated BMDCs also proved the prominent DC activation function of AuNP@HMV@SPs.

### 3.3 In vivo biocompatibility of AuNP@HMV@SPs

**FIGURE 3:**
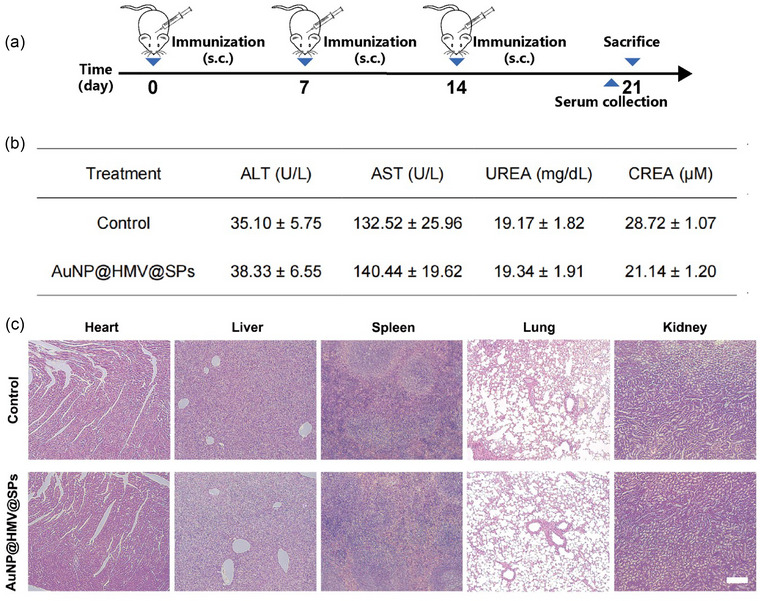
In vivo biocompatibility of AuNP@HMV@SPs. (a) Schematic illustration of experimental design to evaluate the in vivo biocompatibility of AuNP@HMV@SPs (OMVs: 2 µg/mL, SP: 1.5 µg/mL) through subcutaneous injection. Mice without immunization were used as the control group. (b) Biochemical analysis of mouse serum at day 21 (*n* = 4). Normal ranges: ALT (alanine transaminase): 10.06–96.47 U/L, AST (aspartate transaminase): 36.31–235.48 U/L, UREA (blood urea nitrogen): 10.81–34.74 mg/dL, CREA (creatinine): 10.91–85.09 µM. (c) Histological analysis of heart, liver, lung, spleen, and kidney tissues. Scale bar = 200 µm.

The in vivo biocompatibility of AuNP@HMV@SPs was evaluated prior to investigating in vivo immunological activity. Firstly, ICR mice were immunized subcutaneously with AuNP@HMV@SPs on day 0, followed by booster doses on day 7 and day 14. On day 21, various samples were collected for biocompatibility assays (Figure 3a). Blood analysis showed that all the biochemical parameters including alkaline phosphatase (ALT), aspartate aminotransferase (AST), blood urea nitrogen (UREA), and creatinine (CREA) in AuNP@HMV@SPs‐treated mice were in the normal ranges (Figure 3b). Histological analysis of heart, liver, lung, spleen, and kidney tissues was conducted by Hematoxylin and Eosin staining (H&E staining) (Figure 3c). No significant difference between the AuNP@HMV@SPs‐treated group and the control group was observed in H&E‐stained images, indicating the excellent biocompatibility of AuNP@HMV@SPs.

### 3.4 In vivo immune responses activated by AuNP@HMV@SPs

**FIGURE 4:**
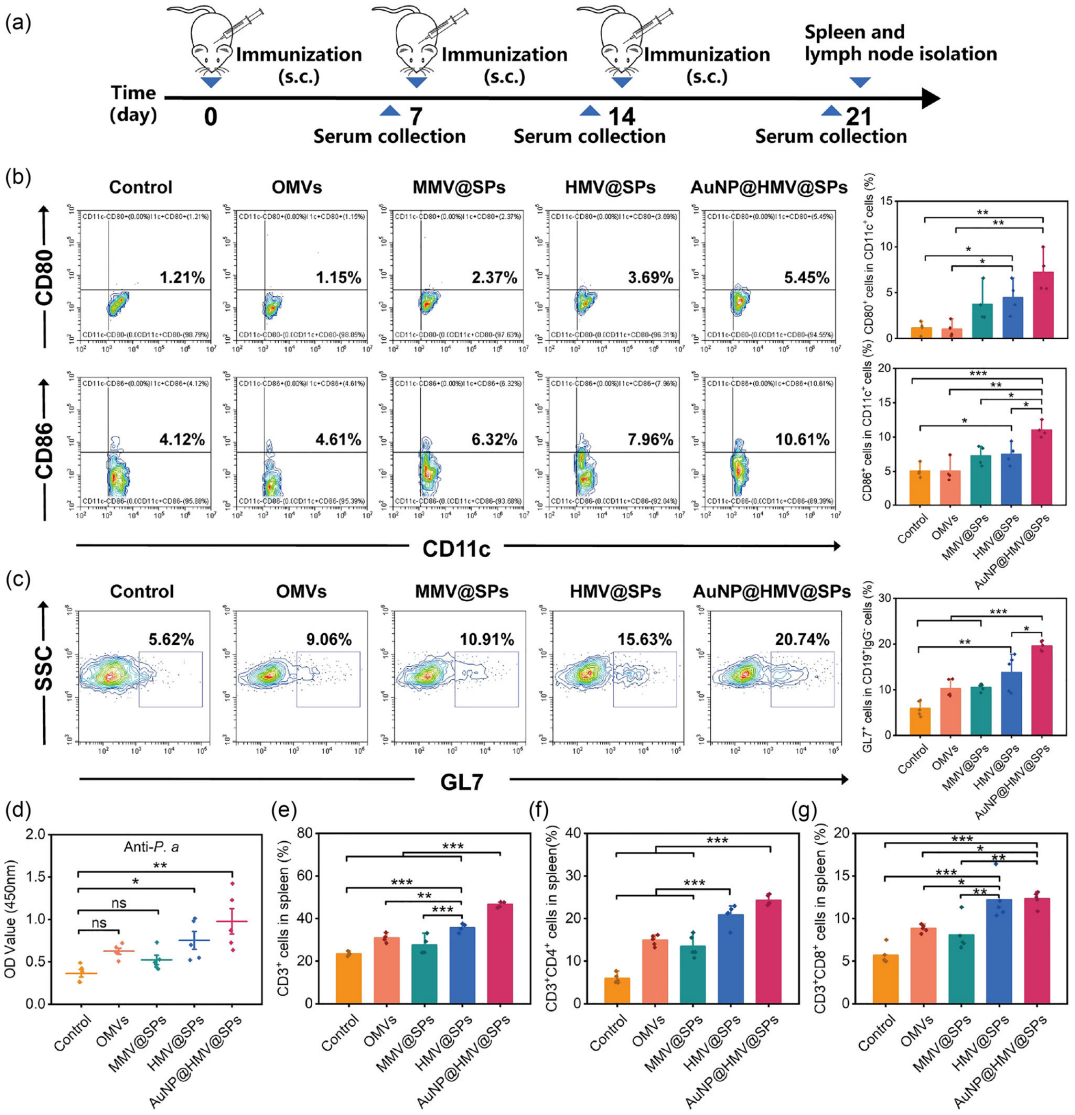
In vivo immune responses activated by AuNP@HMV@SPs. (a) Schematic illustration of experimental design to evaluate the in vivo immune responses activated by AuNP@HMV@SPs (OMVs: 2 µg/mL, SP: 1.5 µg/mL) via subcutaneous injection. (b) Percentage of CD80^+^ and CD86^+^ cells (gated on CD11c^+^ cells) in the lymph nodes collected on day 21 (*n* = 4). (c) Percentage of GL7^+^ cells (gated on CD19^+^IgG^−^ cells) in the spleen collected on day 21 (*n* = 5). (d) Anti‐*P. aeruginosa* specific antibody IgG titers in serum collected on day 21 (*n* = 5). (e) CD3^+^ T cells, (f) CD3^+^CD4^+^ T cells, and (g) CD3^+^CD8^+^ T cells in the spleen collected on day 21 (*n* = 5). Naive mice without immunization were used as control. Statistical analysis was conducted using the student's *t*‐test or One‐way ANOVA with Tukey's post hoc test. *p < 0.05, **p < 0.01, ***p < 0.001 and ns representing non‐significance.

The in vivo immune responses of mice were assessed by subcutaneous injection of AuNP@HMV@SPs on days 0, 7 and 14, followed by the collection of serums, lymph nodes, and spleen on day 21 for analysis (Figure 4a). Since dendritic cells (DCs) would be mature after the uptake of antigens and then migrate to the lymph nodes for further specific immune activation, we examined the frequency of mature DCs expressing co‐stimulatory molecules (CD80 and CD86) in lymph nodes. Consistent with the results of in vitro BMDCs activation, AuNP@HMV@SPs induced the highest frequencies of CD80^+^ and CD86^+^ DCs (CD11c^+^ cells) in the lymph nodes among all treatment groups (Figure 4b). This finding suggested that the co‐delivery of OMVs, SP, and adjuvant promoted the maturation of DCs from an immature to a mature phenotype, which is an essential step for inducing adaptive immune responses.

To confirm the activation of B cell responses, the population of germinal center B cells (GL7^+^ in CD19^+^IgG^−^ cells) in the spleen was examined (**Figure** **4c**). After HMV@SPs vaccination, the proportions of germinal center B cells increased from 6% to 14%, which was higher than the OMVs‐treated group (10%) and MMV@SPs‐treated group (10%). These results indicated that the integration of bacterial membrane and toxins induced a stronger activation of B cells. Moreover, AuNP@HMV@SPs further increased the percentage of germinal center B cells (20%) compared to HMV@SPs, likely due to the small size and stable structure of AuNP@HMV@SPs. The titer of *P. aeruginosa*‐specific antibodies in the serum rose over time after immunization with AuNP@HMV@SPs (Figure S7). Compared with OMVs‐treated mice and MMV@SPs‐treated mice, the titer of *P. aeruginosa*‐specific antibodies, OMVs‐specific antibodies and SP‐specific antibodies in the AuNP@HMV@SPs‐treated mice on day 21 were significantly higher (Figure 4d, S8 and S9). The boost of antibody titers in AuNP@HMV@SPs‐treated mice also suggested the induction of bacterium‐specific B cell responses.

To evaluate the activation of T cell responses, the frequencies of CD4^+^ T cells and CD8^+^ T cells in the spleen were examined (Figure 4e‐g and S10). After immunized with AuNP@HMV@SPs, the proportion of CD4^+^ T cells increased from 6% to 24%, higher than that of the OMVs‐treated group (15%) and MMV@SPs‐treated group (13%). The proportion of CD8^+^ T cells also increased from 6% to 12%, higher than that of the OMVs‐treated group (9%) and MMV@SPs‐treated group (8%). These results implied that AuNP@HMV@SPs increased the population of T cells in spleen to activate specific cellular immunity.

### 3.5 In vivo prophylactic effects in a systemic infection mouse model

**FIGURE 5:**
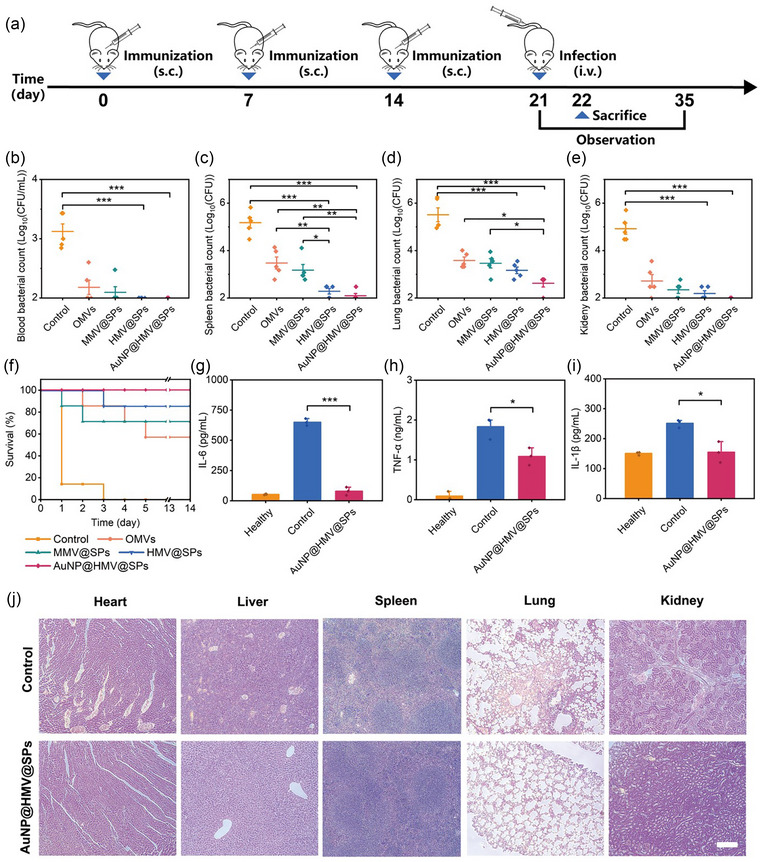
In vivo prophylactic effect of AuNP@HMV@SPs in a systemic *P. aeruginosa* infection model. (a) Schematic illustration of the experimental design to evaluate the in vivo prophylactic effect of AuNP@HMV@SPs (OMVs: 2 µg/mL, SP: 1.5 µg/mL) via subcutaneous injection against systemic *P. aeruginosa* infection. The number of bacteria in (b) blood, (c) spleen, (d) lung, and (e) kidney of the infected mice after different treatments (*n* = 5). (f) Survival curves of the mice intravenously infected with *P. aeruginosa* (2×10^7^ CFU/20 g) after different treatment (*n *= 7). Levels of inflammatory cytokines including (g) IL‐6, (h) TNF‐α, and (i) IL‐1β in the serum of the infected mice after different treatments (*n* = 3). (j) Histological analysis of heart, liver, lung, spleen and kidney tissues of the infected mice after different treatments. Scale bar: 200 µm. Infected mice without any treatment were used as control. Statistical analysis was conducted using the student's *t*‐test or One‐way ANOVA with Tukey's post hoc test. *p < 0.05, **p < 0.01, ***p < 0.001.

The prophylactic effects of AuNP@HMV@SPs in a systemic infection mouse model were evaluated after demonstrating their ability to activate specific in vivo humoral and cellular immunity. A septicemia model was established via intravenous injection of *P. aeruginosa* at day 7 after three‐dose immunization (Figure 5a, S11 and S12). By comparing the number of bacteria present in various tissues among different treatment groups, it was observed that AuNP@HMV@SPs treatment achieved almost complete bacterial clearance in the blood, spleen, and kidney (Figure 5b‐e). Additionally, AuNP@HMV@SPs treatment exhibited 99.9% reduction in bacterial count in the lungs, which was a 10‐fold improvement compared to OMVs and MMV@SPs treatments (Figure 5d). The survival rate of mice was recorded every 24 h (Figure 5f and S13). While the control group exhibited a mortality rate of 60% within 2 days of systemic *P. aeruginosa* infection (1 × 10^7^ CFU/20 g) or a mortality rate of 100% within 3 days of a more severe infection (2 × 10^7^ CFU/20 g), all the mice treated with AuNP@HMV@SPs survived, indicating complete protection of AuNP@HMV@SPs vaccine against systemic *P. aeruginosa* infection. However, in the OMVs‐treated group and MMV@SPs‐treated group, mortality rates of 43% and 29% were observed, respectively, in the case of severe infection (2 × 10^7^ CFU/20 g), suggesting that the immune responses activated by OMVs or SP alone were not potent enough to protect the mice from severe septicemia. Since *P. aeruginosa* infection can induce hyperinflammatory responses and subsequent toxicity, the levels of inflammatory cytokines such as IL‐6, TNF‐α and IL‐1β in the serum were measured (Figure 5g‐i). Treatment with AuNP@HMV@SPs significantly reduced the levels of inflammatory cytokines as compared to the control group, which might be attributed to the clearance of bacteria and toxins by the pre‐activated immune system. Furthermore, histological analysis of tissues including heart, liver, spleen, lung, and kidney revealed that treatment with AuNP@HMV@SPs prevented tissue injury caused by the bacterial infection (Figure 5j).

### 3.6 In vivo prophylactic effects in a pneumonia mouse model

**FIGURE 6:**
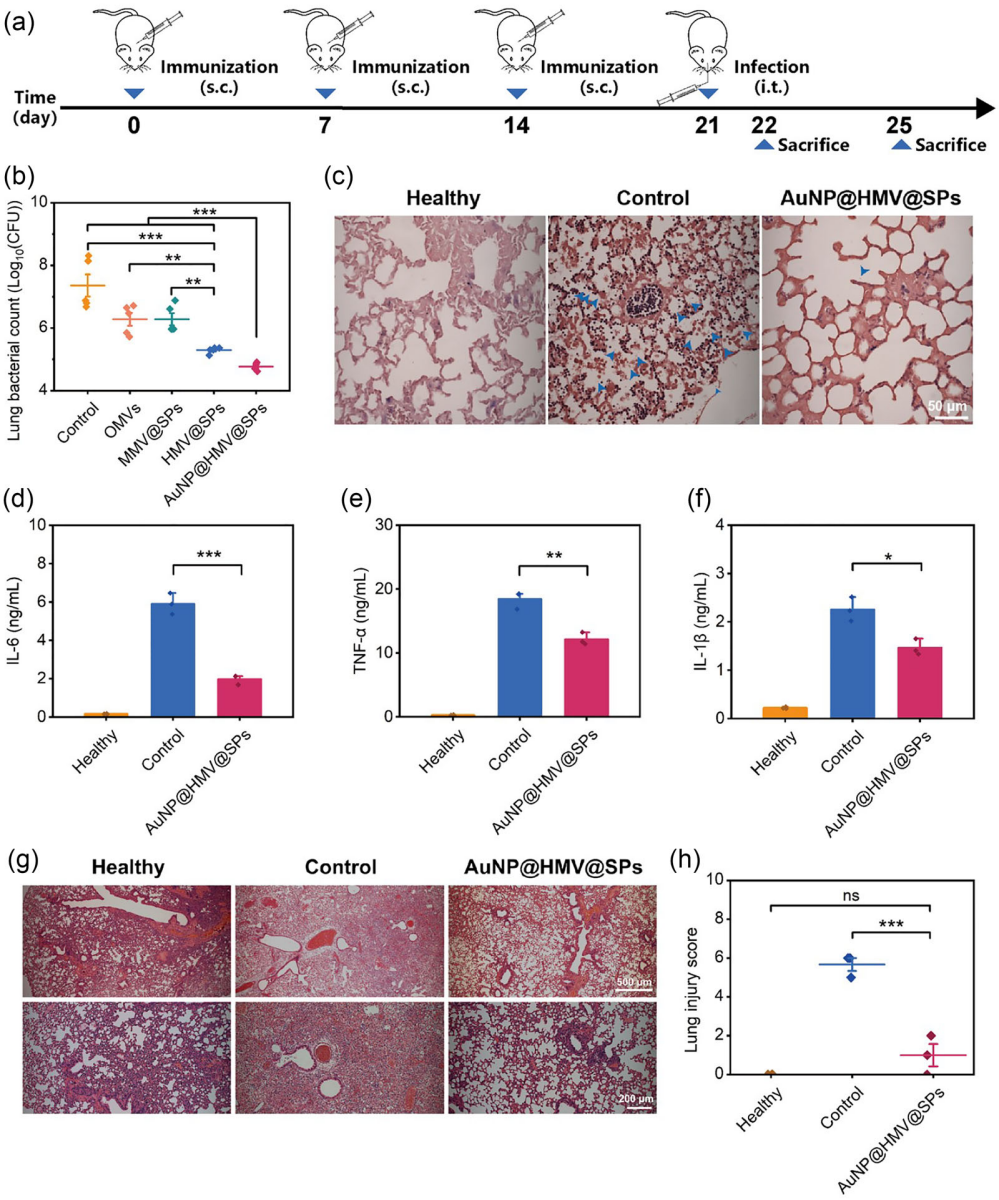
Prophylactic effects of AuNP@HMV@SPs in a pneumonia mouse model. (a) Schematic illustration of the experimental design to evaluate the in vivo prophylactic effects of AuNP@HMV@SPs (OMVs: 2 µg/mL, SP: 1.5 µg/mL) via subcutaneous injection against pneumonia. (b) The number of bacteria in the lungs of the mice immunized with different vaccines after one day after infection (*n* = 5). (c) Gram staining of lung tissue of the immunized mice one day after infection. Scale bar: 50 µm. Blue arrows point to bacteria. Levels of inflammatory cytokines including (d) IL‐6, (e) TNF‐α, and (f) IL‐1β in the serum of the immunized mice one day after infection (*n* = 3). (g) Histological analysis of lung tissue using H&E from the immunized mice four days after infection. Infected mice without vaccination were used as the control group. Scale bar: 500 µm (upper panel) or 200 µm (lower panel). (h) Lung injury score analyzed by the H&E staining images in (g) (*n* = 3). Statistical analysis was conducted using the student's *t*‐test or One‐way ANOVA with Tukey's post hoc test. *p < 0.05, **p < 0.01, ***p < 0.001 and ns representing non‐significance.

The prophylactic efficacy of AuNP@HMV@SPs in a pneumonia mouse model was investigated. A pneumonia model was established via intratracheally administrating *P. aeruginosa* at day 7 after a three‐dose immunization (Figure 6a). The bacteria count in the lungs of mice immunized with HMV@SPs was greatly reduced by approximately 10 folds as compared to the OMVs‐immunized group and MMV@SPs‐immunized group. This result indicated that the co‐delivery of bacterial membrane antigens and toxins enhanced the prophylactic efficacy of the anti‐*P. aeruginosa* vaccine (Figure 6b). More importantly, the vaccination with AuNP@HMV@SPs exhibited the highest antibacterial potency with approximately 99.9% bacterial reduction in the lungs, as confirmed by the lung Gram staining images (Figure 6b and 6c). The improved protection could be attributed to the strong specific immune responses activated by AuNP@HMV@SPs (Figure 4). The significantly decreased level of inflammatory cytokines such as IL‐6, TNF‐α, and IL‐1β in the lungs of mice immunized with AuNP@HMV@SPs suggested that the vaccine was able to inhibit hyperinflammatory responses in pneumonia (Figure 6d‐f). The histological analysis of the lungs using H&E staining further confirmed that the mice vaccinated with AuNP@HMV@SPs effectively prevented lung injury in the pneumonia model, as indicated by the significantly lower lung injury score (Figure 6g and h).

## 4 CONCLUSION

We have developed a bacterial‐macrophage hybrid cell membrane‐based vaccine (AuNP@HMV@SPs) for prevention of bacterial infections. This vaccine, which contains enriched bacterial antigens from bacterial cell membrane and secreted toxins, was proposed for the prevention of *P. aeruginosa* infections. The incorporation of macrophage membrane in AuNP@HMV@SPs significantly reduced the inflammatory toxicity, hemolysis and cytotoxicity associated with OMVs and exotoxin, resulting in improved compatibility both in vitro and in vivo. Moreover, the inclusion of AuNPs as an adjuvant promotes maturation of dendritic cells and activation of specific humoral and cellular immunity. By presenting multiple bacterial antigens on the surface, along with AuNPs as adjuvant, AuNP@HMV@SPs demonstrated high prophylactic efficacy against *P. auruginosa*‐induced septicemia with 100% mouse survival, and attenuation of infection severity in a pneumonia model. Overall, these hybrid cell membrane‐functionalized nanovaccine provide a safe and effective strategy for preventing *P. aeruginosa* infection by eliciting enhanced specific immune responses for bacterial cell killing and toxin neutralization.

The strategy of using nanovaccines to co‐deliver bacterial membrane antigens and secreted toxins offers a promising approach to addressing a wide range of drug‐resistant bacterial infections, potentially overcoming the limitations of traditional vaccines. However, the use of mouse‐derived macrophages in our current study may restrict the clinical translation of these vaccines. Future research should focus on utilizing human‐derived macrophages to enhance the relevance and applicability of the vaccine. Additionally, incorporating bacteria‐activated macrophages could improve antigen binding affinity, further boosting vaccine efficacy. Comprehensive analysis of the protein profile and composition of the nanovaccine is essential to understand their biological effects fully. Such insights could lead to the development of more effective vaccines.

## AUTHOR CONTRIBUTIONS

Xinran Peng and Xin Ding conceived the concept. Xinran Peng and Xin Ding designed experiments and write the manuscript. Xinran Peng performed most of experiments and analyzed data. Yuanjing Luo and Li Yang involved in the antibacterial experiments of in vivo study. Xinhai Chen and Guo‐Bao Tian involved in discussion in experiment design and reviewed the manuscript. Yi Yan Yang, Peiyan Yuan, and Xin Ding supervised and/or provided funding to this project, reviewed and edited the manuscript. All authors have read and approved the manuscript.

## CONFLICT OF INTEREST STATEMENT

The authors declare no conflicts of interest.

## ETHICS APPROVAL STATEMENT

All the in vivo experiments performed were approved by the Institutional Animal Care and Use Committee of School of Pharmaceutical Sciences (Shenzhen), Sun Yat‐sen University (Approval No. SYSU‐YXYSZ‐20220308) and Guangzhou Institutes of Biomedicine and Health (GIBH), Chinese Academy of Science (Approval No. N2022138).

## Supporting information



Supporting Information

Supporting Information

## Data Availability

The datasets during and/or analysed during the current study available from the corresponding author on reasonable request.
