# Supplementary material for: A multiantigenic antibacterial nanovaccine utilizing hybrid membrane vesicles for combating Pseudomonas aeruginosa infections
Source: J Extracell Vesicles. 2024 Oct 14;13(10):e12524. doi: 10.1002/jev2.12524 (PMC11472236; doi:10.1002/jev2.12524)
Supplement: Supplementary file 1 — Supporting Information [file JEV2-13-e12524-s001.pdf]

Supporting Information

A Multiantigenic Antibacterial Nanovaccine Utilizing Hybrid Membrane Vesicles for Combating *Pseudomonas aeruginosa* Infections

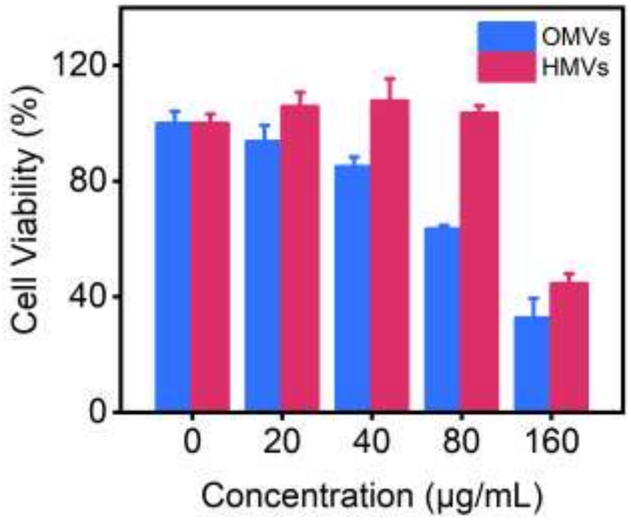

Figure S1. Viability of RAW 264.7 cells after incubating RAW 264.7 cells with OMVs or HMVs at various protein concentration of OMVs for 24 h (protein weight ratio of OMVs to MMVs is 1:2) (n = 3).

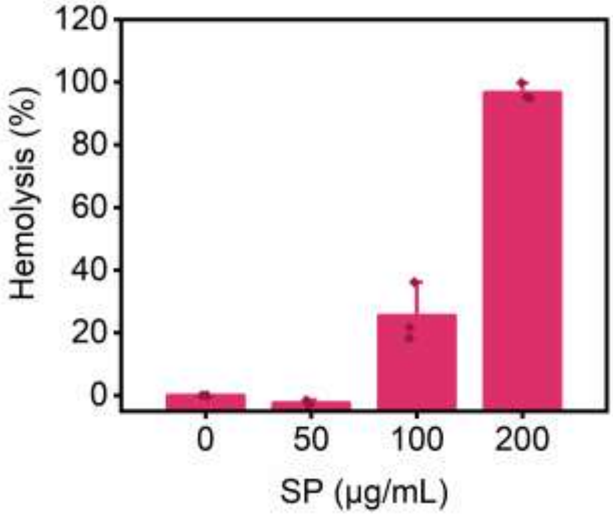

Figure S2. Hemolytic activity of SP at various concentrations after incubation with mouse red blood cells for 0.5 h (n = 3).

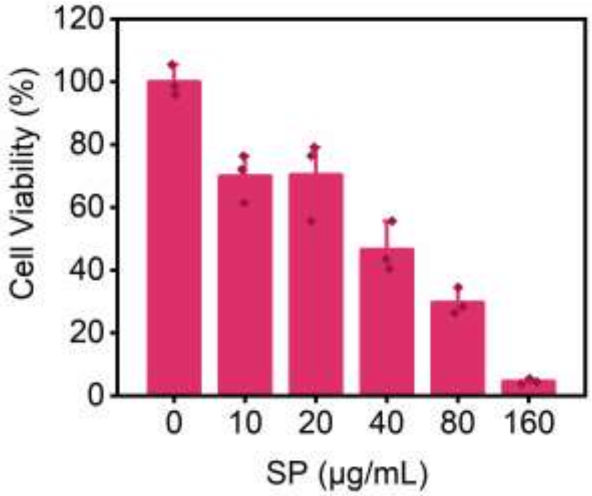

Figure S3. Viability of RAW 264.7 cells after incubation with SP at various concentrations for 24 h (n = 3).

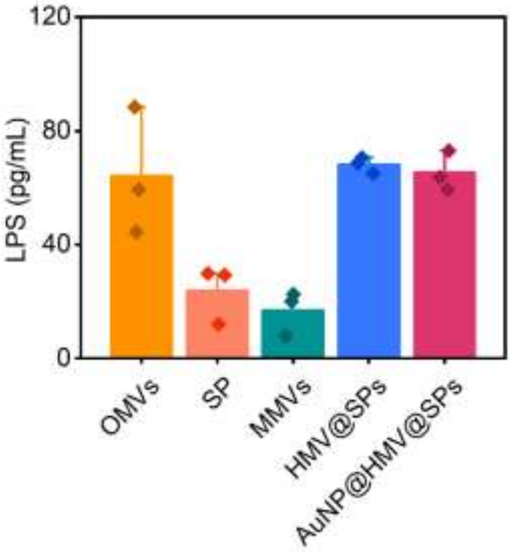

Figure S4. LPS content of OMVs, SP, MMVs, HMV@SPs and AuNP@HMV@SPs measured by ELISA assay (OMVs: 2 μg, SP: 1.5 μg, MMVs: 4 μg, n = 3).

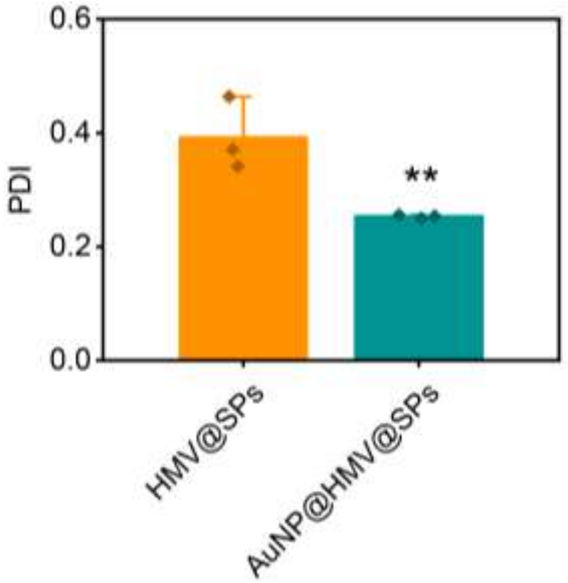

Figure S5. Polydispersity index (PDI) of HMV@SPs and AuNP@HMV@SPs.

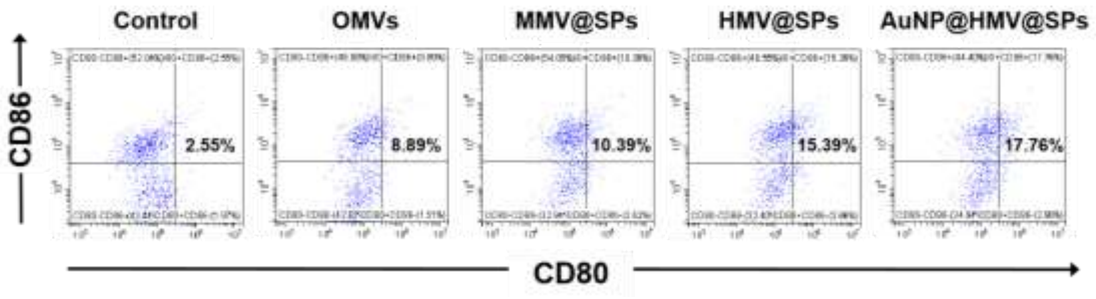

Figure S6. Expression levels of CD80^{+}CD86^{+} in BMDCs (CD11c^{+}) after different treatments *in vitro* for 24 h.

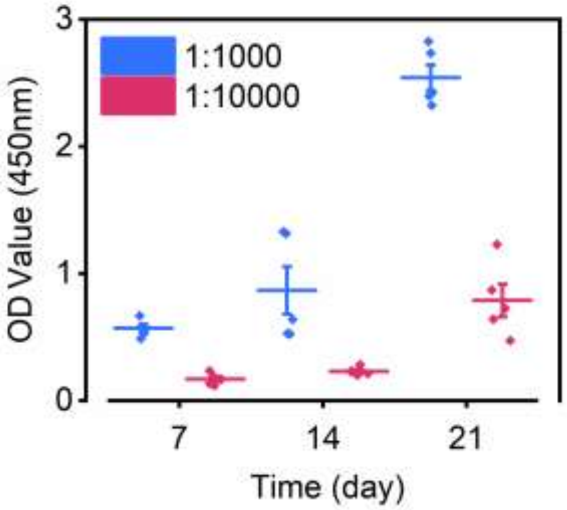

Figure S7. Anti-*P. aeruginosa* specific antibody IgG titers in the serum collected on day 7, day 14 and day 21 after subcutaneous injection of AuNP@HMV@SPs (2 µg OMVs and 1.5 µg SP). The serum samples were diluted by 1000 or 10000 times for evaluation of IgG titers. (n = 5).

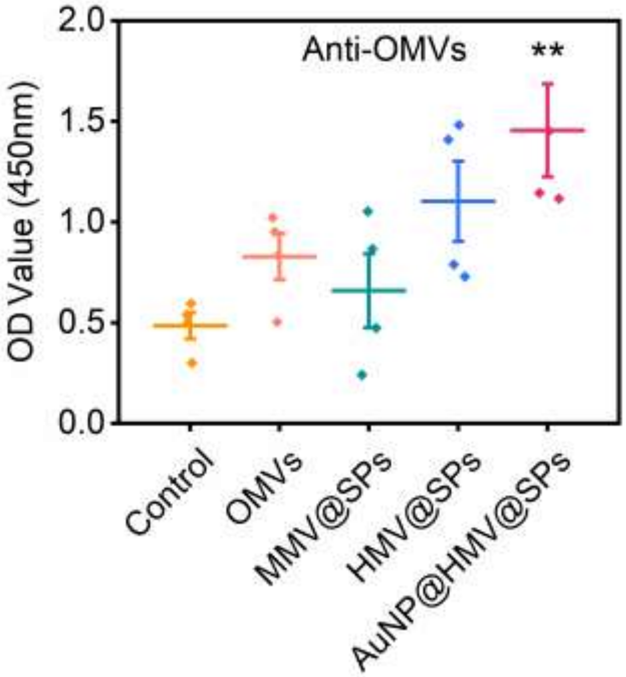

Figure S8. Anti-OMVs specific antibody IgG titers in the serum collected on day 21 after subcutaneous injection of AuNP@HMV@SPs (OMVs: 2 µg/mL, SP: 1.5 µg/mL) (n = 4).

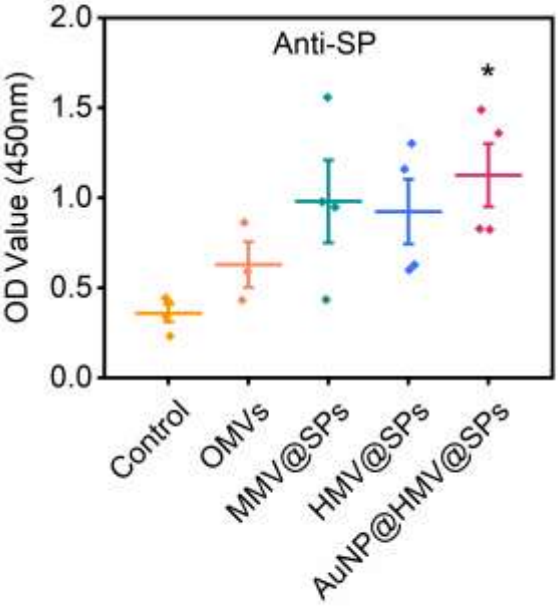

Figure S9. Anti-SP specific antibody IgG titers in the serum collected on day 21 after subcutaneous injection of AuNP@HMV@SPs (2 μg OMVs and 1.5 μg SP) (n = 4).

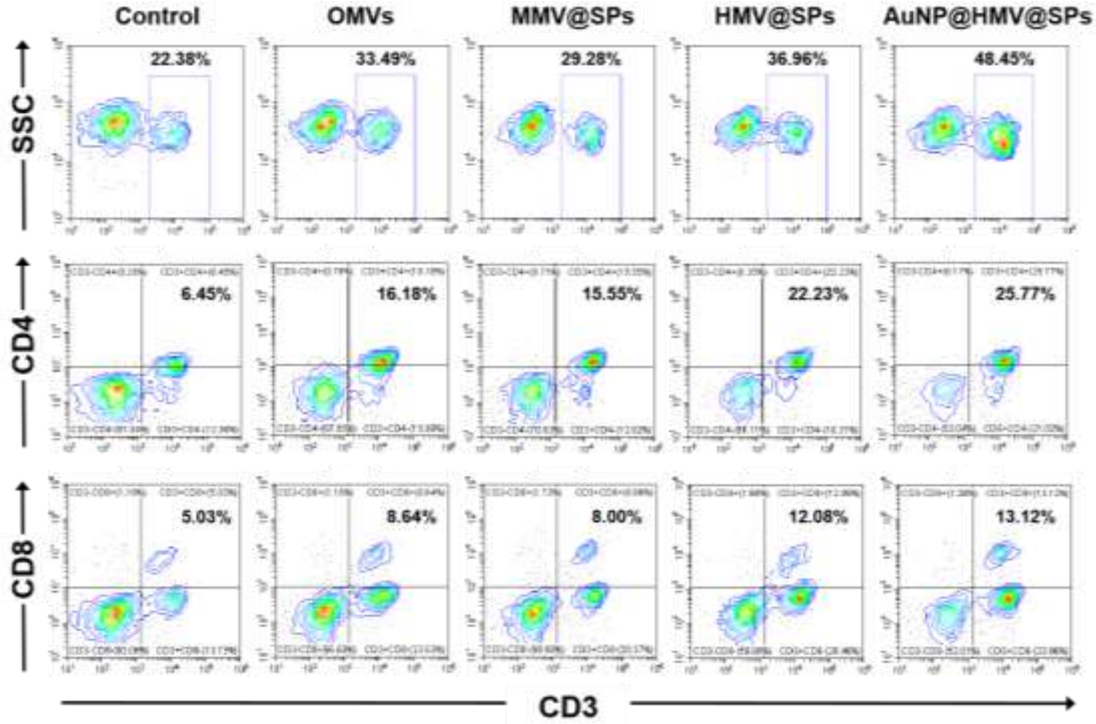

Figure S10. Percentage of $CD3^+$, $CD3^+CD4^+$ and $CD3^+CD8^+$ T cells in the spleen characterized by flow cytometry on day 21 after subcutaneous injection of AuNP@HMV@SPs (OMVs: 2 μg/mL, SP: 1.5 μg/mL).

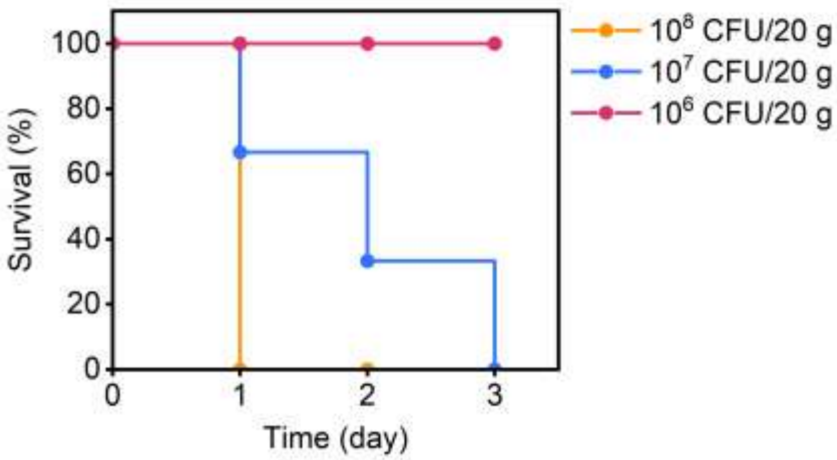

Figure S11. Survival rate of the mice intravenously infected with *P. aeruginosa* at different doses (n = 3).

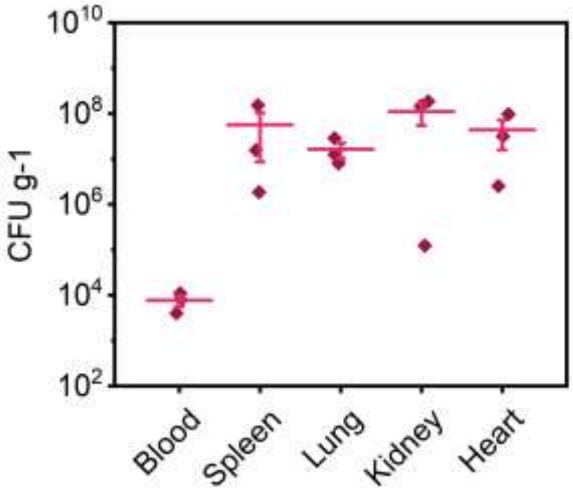

Figure S12. Number of bacterial counts in blood, spleen, lung kidney and hear of the infected mice after intravenous infection with *P. aeruginosa* ($1×10^7$ CFU/20 g, n = 3). The unit of Y-axis for the blood sample: CFU/mL.

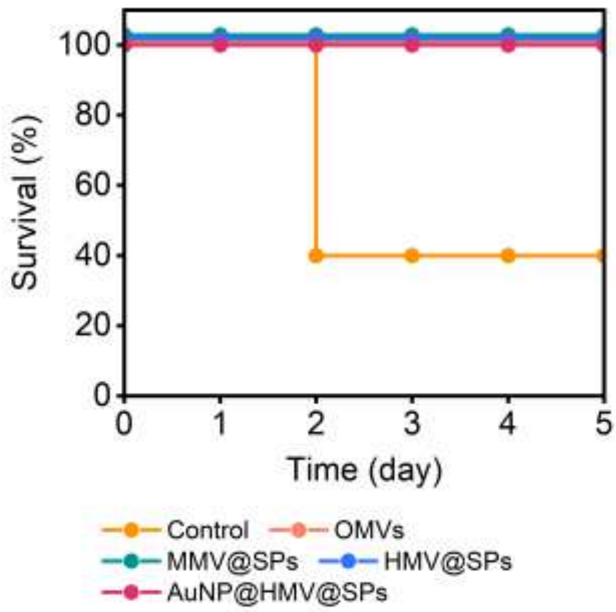

Figure S13. Survival rate of the mice intravenously infected with *P. aeruginosa* (1×10^7 CFU/20 g) after different treatments (n = 5).

Table S1. Antibiogram of *P. aeruginosa* (PA17ZC010).

Antibiotic agents / organisms	MIC (μg/mL)
Piperacillin	16
Ceftazidime	8
Cefepime	16
Aztreonam	16
Piperacillin/Taz	32/4
Imipenem	2
Meropenem	1
Ciprofloxacin	0.5
Levofloxacin	1
Gentamicin	2
Amikacin	8
Colistin	1
